# Relationship chains of subhealth physical examination indicators: a cross-sectional study using the PLS-SEM approach

**DOI:** 10.1038/s41598-023-39934-5

**Published:** 2023-08-22

**Authors:** Yu Wang, Jindi Lou, Jun Li, Yulin Shi, Tao Jiang, Liping Tu, Jiatuo Xu

**Affiliations:** 1 School of Public Health, Shanghai University of Traditional Chinese Medicine, 1200 Cailun Road, Pudong, Shanghai, China; 2 School of Traditional Chinese Medicine, Shanghai University of Traditional Chinese Medicine, 1200 Cailun Road, Pudong, Shanghai, China; 3 Experiment Center For Teaching and Learning, Shanghai University of Traditional Chinese Medicine, 1200 Cailun Road, Pudong, Shanghai, China

**Keywords:** Physical examination, Signs and symptoms, Oral manifestations

## Abstract

Subhealth is a transitional state between health and disease, and it can be detected through routine physical check-ups. However, the complexity and diversity of physical examination items and the difficulty of quantifying subhealth manifestations are the main problems that hinder its treatment. The aim of this study was to systematically investigate the physical examination performance of the subhealthy population and further explore the deeper relationships between indicators. Indicators were obtained for 878 subjects, including basic information, Western medicine indicators, inquiries of traditional Chinese medicine and sublingual vein (SV) characteristics. Statistical differences were analysed using R software. To explore the distribution of symptoms and symptom clusters in subhealth, partial least squares-structural equation modelling (PLS-SEM) was applied to the subhealth physical examination index, and a structural model was developed to verify whether the relationship chain between the latent variables was reasonable. Finally, the reliability and validity of the PLS-SE model were assessed. The most common subclinical clinical symptoms were limb soreness (37.6%), fatigue (31.6%), shoulder and neck pain (30.5%) and dry eyes (29.2%). The redness of the SV in the subhealthy group was paler than that in the healthy group (*p* < 0.001). This study validates the establishment of the directed acyclic relationship chain in the subhealthy group: the path from routine blood tests to lipid metabolism (t = 7.878, *p* < 0.001), the path from lipid metabolism to obesity (t = 8.410, *p* < 0.001), the path from obesity to SV characteristics (t = 2.237, *p* = 0.025), and the path from liver function to SV characteristics (t = 2.215, *p* = 0.027). The innovative application of PLS-SEM to the study of subhealth has revealed the existence of a chain of relationships between physical examination indicators, which will provide a basis for further exploration of subhealth mechanisms and causal inference. This study has identified the typical symptoms of subhealth, and their early management will help to advance the treatment of diseases.

## Introduction

In recent years, the concept of subhealth has been widely accepted in many countries, such as China, Japan, Canada and Australia^1–3^. The global incidence of subhealth was reported to be between 57.2 and 65.1%^4,5^. With reference to the World Health Organization's definition of health, subhealth is defined as “the third state” with functional changes but no organic pathological changes^6^. Subhealth is a state characterized by some disturbances in psychological behaviours or physical characteristics or in some indices of medical examination^7^. Specifically, physical discomfort related to subhealth is mainly characterized by physical symptoms such as fatigue, sleep disorders, or pain. Psychological discomfort of subhealth is mainly characterized by depression or restlessness, irritability, fear and timidity, memory decline, inability to concentrate and other mental symptoms^8^. TCM clinical guidelines for subhealth released by the China Association of Chinese Medicine pointed out that when the abovementioned state persists for more than 3 months and diseases that may cause the abovementioned manifestations are excluded by systematic examination, the person is judged to be in a state of subhealth^9^. In summary, as a transitional state between health and disease, the judgement subhealth is mainly based on clinical symptoms. If patterns of subhealth symptoms are identified, it will help to intervene in poor health behaviours and implement disease prevention. Therefore, what are the most typical symptoms of subhealth?

Routine physical examination is an effective way to analyse the objective indicators of the examinee and to comprehensively assess the manifestation of symptoms. The subjects of routine physical examination are mainly healthy and sick people. In fact, routine physical examinations, an extremely common method of primary care for adults^10,11^, are not only applicable to healthy and sick people but are also a major means of identifying and preventing subhealth. However, due to the complexity of physical examination indicators and the difficulty of quantifying clinical symptoms, few studies have been conducted to systematically investigate the routine physical examinations of the subhealthy population and further explore the deeper relationships between subhealth indicators. For example, what are the associations between complex physical examination items such as morphology, liver function, blood count, etc.

Partial least squares-structural equation modelling (PLS-SEM) provides a way to do this: it can assume the existence of a chain of relationships between the above physical examination items, build a conceptual relationship model, and test statistically whether the model is robust. Notably, given the flexibility of the PLS-SEM technique in terms of data allocation and its suitability for small sample sizes^12^, many studies in the fields of clinical medicine and public health have validated and used it in recent years^13–17^. If the PLS-SEM approach can be used to find links between complex and diverse physical examination indicators, it will help to screen for subhealth-specific medical examination programs and reduce health care costs for subhealthy populations. Previously, we studied the oral characteristics of the healthy population and found age-related changes in the colour of the sublingual veins (SV)^18,19^. Therefore, the SV characteristics were also included as one of the physical examination items. Concepts such as “anthropometric indicators”, “laboratory tests” and “SV characteristics” cannot be measured directly and are called “Latent Variables”. However, they can be reflected by a series of specific indicators. For example, haemoglobin (HGB) and red blood cells (RBC) reflect the status of blood operation, total cholesterol (TC) and triglycerides (TG) reflect the level of lipid metabolism, alanine transaminase (ALT) and aspartate transaminase (AST) reflect liver function, and body mass index (BMI) and waist-to-hip ratio (WHR) can be directly measured and show obesity. These specific quantitative indicators are called “Observed Variables”. In brief, PLS-SEM demonstrates the structure of a relational chain in which latent variables can be separately represented by a set of observed variables. Therefore, this study aims to combine TCM diagnostic information to explore the typical manifestations of subhealth and, more importantly, to discover the deep relationships between the complex physical examination indicators of subhealthy populations through the PLS-SEM approach.

## Material and methods

### Study population

**Figure 1:**
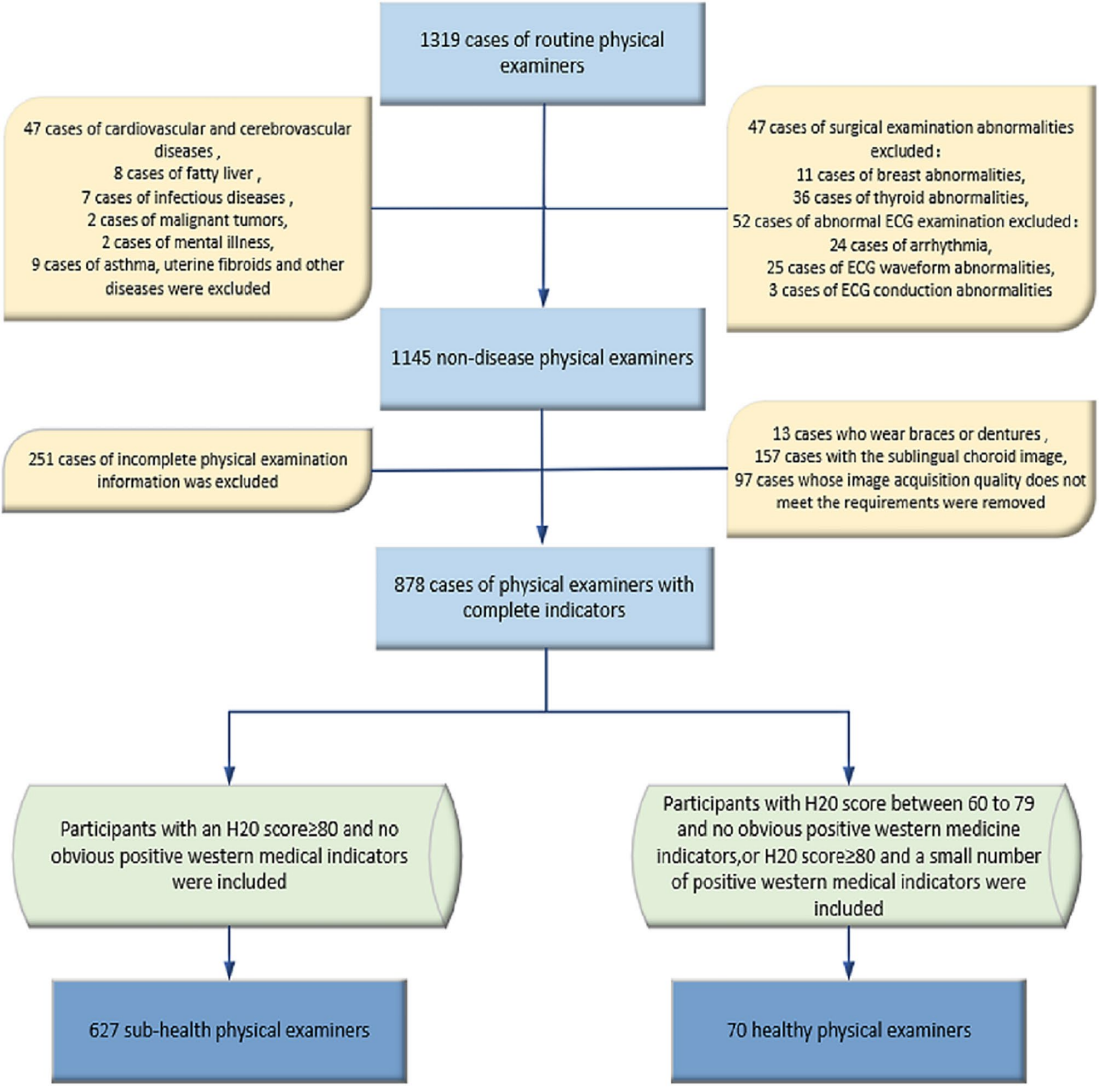
Flowchart demonstrating the source of cross-sectional data and the inclusion process; Shanghai, China, 2020–2021.

This study was designed as a cross-sectional study with 1319 subjects who underwent routine medical examination at the medical examination centre, Shuguang Hospital Affiliated to Shanghai University of Traditional Chinese Medicine, from June 2020 to July 2021. We collected their basic information and assessed laboratory indices and oral SV. A number of trained clinicians instructed the examiners on site to complete the Health Status Assessment Questionnaire (H20), and they interviewed and clinically examined the physical examinees by completing the “Chinese Medicine Four Diagnosis Information Record Form” (Copyright No. 2016Z11L025702) designed by the subhealth research group of the “863 Plan” to investigate the health status of the examiners over three months. “Chinese Medicine Four Diagnosis Information Record Form” is the collection and collation of clinical information using the principles of TCM diagnosis, with the clinician recording the frequency and severity of the subject’s symptoms from low to high on a scale of 0, 1 and 2 (Supplementary Table S1). After rigorous exclusion, 627 subhealthy individuals and 70 healthy individuals were finally included (Fig. 1).

### Selection criteria

Subhealth criteria were as follows: an H20 score of 60–79 with no significant positive Western medicine indicators or an H20 score ≥ 80 with few positive Western medicine indicators. Health criteria were as follows: H20 score ≥ 80 and/or no significant positive Western medicine indicators.

### Physical examination indicators

Information on previous medical history, smoking status and educational level are taken from the medical examination confirmation form. Qualified workers in the Medical Examination Center of Shuguang Hospital followed a standard operating procedure to measure height, weight, waist circumference and hip circumference to calculate BMI and WHR. The clinical Western indices mainly included alanine transaminase (ALT), AST, alkaline phosphatase (AKP), gamma-glutamyl transferase (GGT), TC, TG, low-density lipoprotein (LDL), RBC, HGB, mean corpuscular haemoglobin concentration (MCHC) and red blood cell specific volume (HCT). The above indicators were tested by the Department of Laboratory Medicine of Shuguang Hospital using an automated biochemical analyser (Beckman Coulter AU5800, California, USA).

Inquiry traditional Chinese medicine (TCM) is regarded as “the essentials of diagnosis and the first task of clinical symptoms” by TCM, which refers to the comprehensive collection of disease data obtained through the inquiry of professional clinicians but cannot be obtained by other diagnosis methods^20^. Symptom indices of TCM are the presence and frequency of symptoms related to psyche, cold and heat, sweat, head symptoms, chest and abdomen symptoms, diet, taste, coughing-up phlegm, passing stool, urination, pain, menstruation, and leucorrhoea. They were all derived from the clinician's enquiry, and the results were recorded in the TCM clinical diagnosis record table (Supplementary Table S1).

### Acquisition and analysis of SV

**Figure 2:**
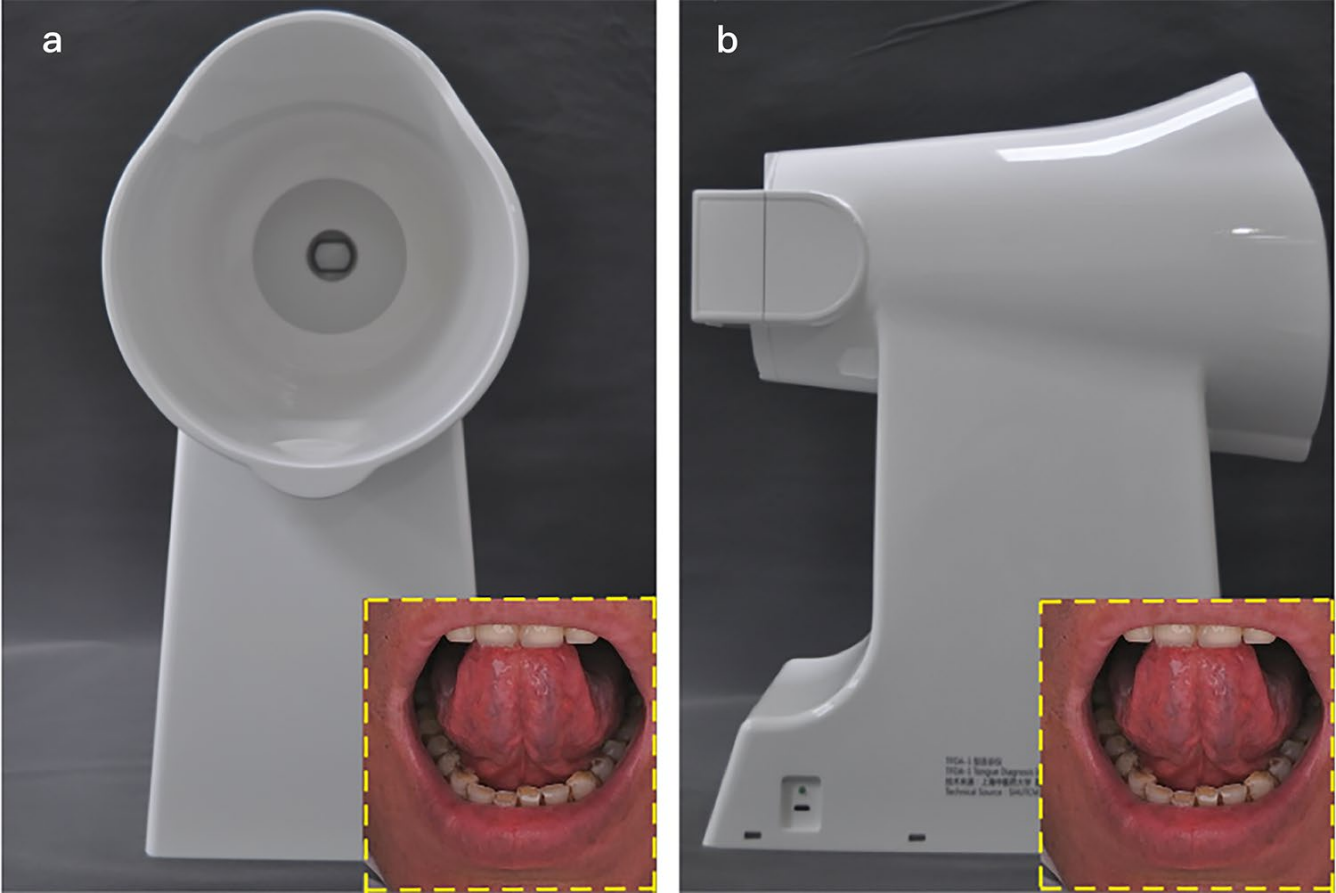
Figures of the TFDA-1 tongue and face diagnosis instrument; Shanghai, China, 2020–2021. (**a**) Front view, (**b**) Profile view.

Researchers in the Laboratory of Intelligent Processing of TCM Diagnostic Information, Shanghai University of Traditional Chinese Medicine, used the self-developed Tongue and Face Diagnosis Instrument-1 (TFDA-1) to collect tongue images (Fig. 2). It is based on the National Key Research and Development Program for Modernization of Chinese Medicine Research Special Project (No. 2017YFC1703301) and has been used for clinical tongue image collection to assess organism status^21–23^. Its D50 light source offers high stability and colour reproduction. It is worth noting that TFDA-1 was specially equipped with a lower jaw rest to hold the lower jaw to ensure the standardized distance between the tongue and lens, thus avoiding distortion of the SV image in early research^24^. The acquisition period was from 8:00 a.m. to 11:00 a.m. Before acquisition, the instrument was sterilized by wiping with an alcohol cotton ball, and participants were confirmed to have maintained an empty stomach, clean mouth, and no foreign bodies or abnormal coloring on the tongue. Participants assumed a seated position, put their lower jaw against the lower jaw rest, lightly closed their eyes, opened their mouth and kept their tongue still while lightly pressing the tip of the tongue against the boundary between the upper jaw and the incisors to fully expose the sublingual area.

**Figure 3:**
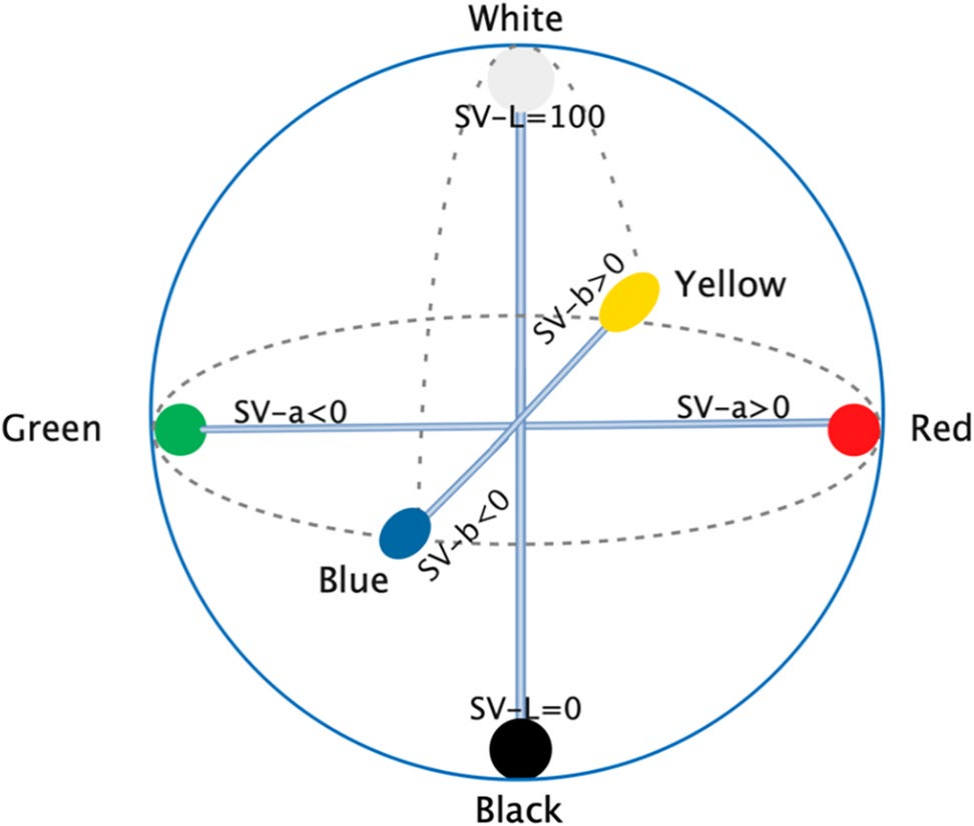
SV-Lab colour space; Shanghai, China, 2020–2021.

Using the Tongue Diagnosis Analysis System (TDAS) (V3.0) (software copyright registration No. 2018SR033451, Shanghai University of TCM, Shanghai, China) developed by the Laboratory of Intelligent Processing of TCM Diagnostic Information, Shanghai University of Traditional Chinese Medicine, RGB colour values of the photographed tongue image pixels were calculated^25^. The colour representation intuitiveness and classification feasibility were taken into consideration when using chromaticity space. The LAB colour model is the most suitable colour model for the modernization of tongue images because it can represent all the colours that can be perceived by human eyes, so the RGB colour space was converted into LAB colour space^26,27^. The tongue colour characteristic indices SV-L, SV-a, and SV-b were derived. SV-L represents lightness, SV-a represents the red‒green axis, and SV-b represents the yellow‒blue axis^28,29^. Specifically, a larger value of SV-L indicates more brightness; SV-a represents the range from red to green, with a higher SV-a value indicating a redder colour and a lower value indicating a greener colour; SV-b represents the range from yellow to blue, and similarly, a lower SV-b value indicates a bluer colour and a higher value indicates more yellow (Fig. 3).

The specific criteria for grading the morphology of SV are shown in Table 1. The tortuosity of the SV was independently and double-blindly determined by three experts with the title of attending physician and more than 5 years of clinical work. Two experts (Cheng and Li) made the diagnosis of tortuosity, and only in cases of disagreement was the third gold standard expert (Tu), the most experienced clinician, consulted to reach a final conclusion. After that, the digital images were preprocessed by LabelMe (V3.16.1, MIT, Massachusetts, USA) for SV morphology, and SV edges were divided into continuous line segments. SV images were imported into Digimizer (V4.2.6.0), MedCalc Software Ltd, Ostend, Belgium) for automatic determination. The scale of digital picture pixels to actual length was experimentally calculated to be 1/177.171, from which the actual values (unit: mm) of the trunk length and width of SV were converted.

**Table 1:** Grading criteria of SV morphology; Shanghai, China, 2020–2021.

Morphology of SV	Grading		
Illustrations			
Tortuosity	T1	T2	T3
	No obvious tortuosity	Tortuous veins or accompanied by prolonged veins	The veins are tortuous and prolonged, with branches like tree branches
Trunk-length	L1	L2	L3
	$\frac{{\mathrm{a}}_{1}+{\mathrm{a}}_{2}}{2}<\frac{3}{5}\times \mathrm{ b}$ The length is shorter than 3/5 of the line between sublingual caruncle and tip of the tongue	$\frac{{\mathrm{a}}_{1}+{\mathrm{a}}_{2}}{2}=\frac{3}{5}\times \mathrm{ b}$ The length is approximately 3/5 of the line between sublingual caruncle and tip of the tongue	$\frac{{\mathrm{a}}_{1}+{\mathrm{a}}_{2}}{2}>\frac{3}{5}\times \mathrm{ b}$ The length is longer than 3/5 of the line between sublingual caruncle and tip of the tongue
Width	W1	W2	W3
	The maximum width of the vein is less than 3 mm	The maximum width of the vein is 3 ~ 4 mm	The maximum width of the vein is more than 4 mm

*SV* sublingual veins.

**Figure 4:**
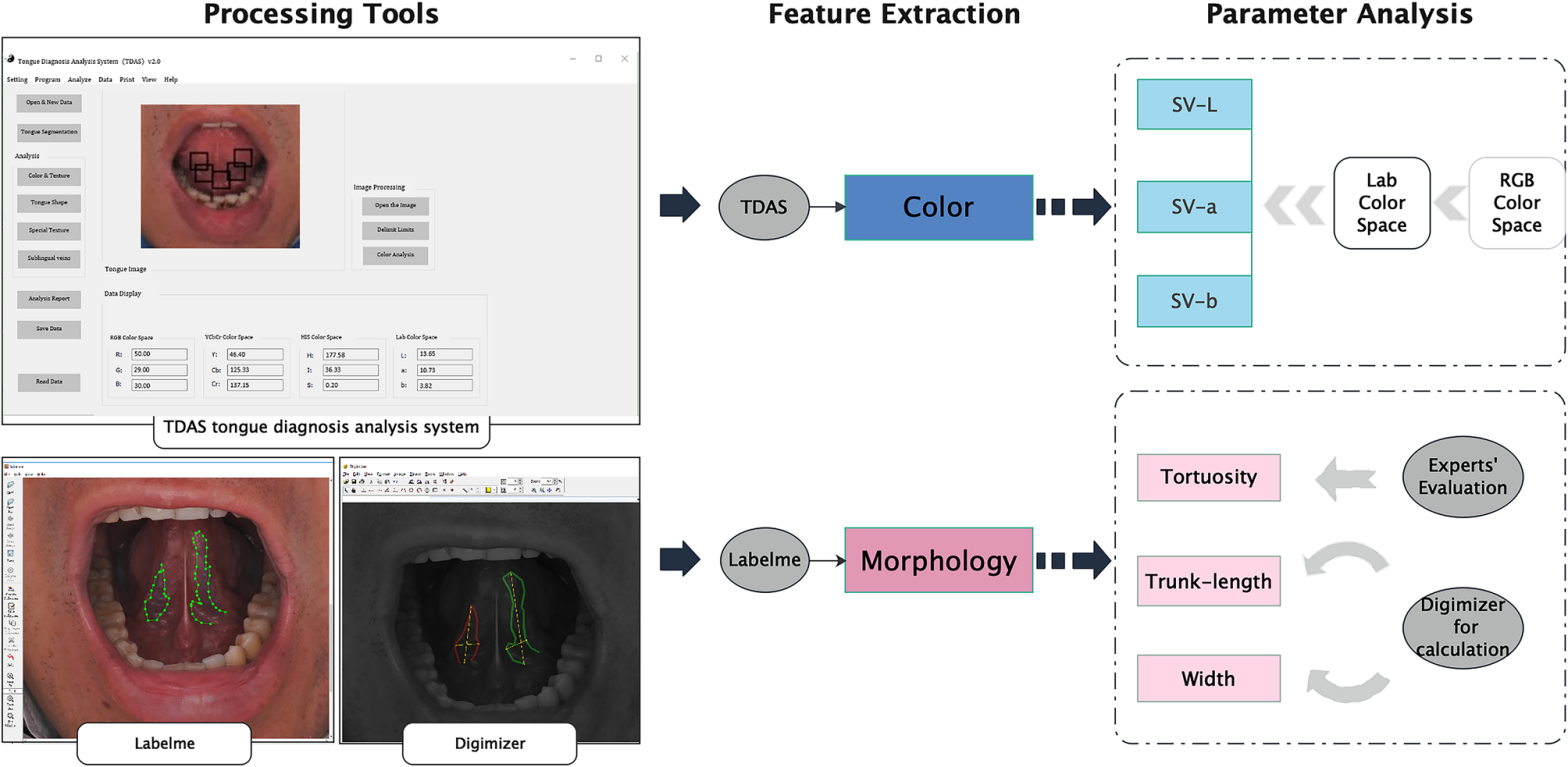
Flow diagram of the analysis of core SV characteristics; Shanghai, China, 2020–2021.

The overall flow diagram of SV characteristics is shown in Fig. 4.

### Statistical analysis

Statistical analysis was performed using R software 4.2.1 (R, Auckland,New Zealand). For the small number of missing values of Western medicine indicators, single imputation was used. First, we analysed the distribution pattern of the data. If it conformed to the normal distribution, the mean could be used for imputation. If it did not conform to the normal distribution, the median was used for imputation. The missing values were eventually filled. For the measurement data that conformed to a normal distribution and were expressed as the mean ± SD, we used two independent-sample t tests to compare two groups. The count data, which were expressed as frequencies and composition ratios, were analysed and tested with the chi-square test and Mann‒Whitney U test. The hypothesis testing was performed using a two-sided α of 0.05 as the significance criterion.

This study applied the PLS-SEM approach to develop the conceptual model and examine the proposed hypotheses using SmartPLS 4.0.8.3. It was performed in two phases. In the first stage, the model’s construct reliability and validity were evaluated^30^. More specifically, Cronbach's alpha, average variance extracted (AVE), and composite reliability (CR) were used to assess the reliability, convergent validity and composite reliability of the reflective measurement model; outer weights and variance inflation factors (VIFs) were used to assess the validity and indicator appropriateness of the formative measurement model^31^. In the second stage, statistical significance testing and path coefficient calculation were performed based on bootstrapping with 5000 resamples. Factor loading was used to denote model convergent validity, while the heterotrait-monotrait ratio (HTMT) and correlation were used to denote discriminant validity.

### Ethics approval and consent to participate

The study was conducted according to the guidelines of the Declaration of Helsinki and was approved by the institutional review board of Shuguang Hospital affiliated with Shanghai University of Traditional Chinese Medicine (2018-626-55-01). All participants signed a written informed consent form that was approved by the Ethics Committee.

## Results

### Basic information of the physical examinees

By investigating the demographic data and laboratory indicators of the physical examinees, we found that the subhealthy people were younger in this cross-sectional study, with a mean age of 33.41 ± 8.85. The proportion of smoking among subhealthy physical examinees was also higher, accounting for 20.6%. The educational level of the total medical examination population was mainly at the level of a university degree. It could be seen from the laboratory that the average value of each index was in accordance with the normal range. The above information is shown in Table 2.

**Table 2:** Basic characteristics of the physical examinees at the medical examination centre; Shanghai, China, 2020–2021.

Variables	Subhealthy (n = 627)		Healthy (n = 70)		*P* value
	N/Mean	%/SD	N/Mean	%/SD	
Age (years), mean (SD)	33.41	8.85	38.07	11.6	< 0.001^a^
WHR, mean (SD)	0.84	0.08	0.85	0.18	0.254^a^
BMI (kg/m^2^), mean (SD)	23.71	3.65	22.67	2.93	0.023^a^
Sex, N (%)					0.004^b^
Male	316	50.4	22	31.4	
Female	311	49.6	48	68.6	
Smoking status, N (%)					0.275^b^
Never smoked	498	79.4	60	85.7	
Smoker	129	20.6	10	14.3	
Educational level, N (%)					0.022^b^
Graduate student	130	21.1	6	8.6	
University student	393	63.8	46	65.7	
Secondary school students	27	4.4	6	8.6	
High school and below or other	66	10.7	12	17.1	
AST (U/L), mean (SD)	22.03	8.7	20.07	4.36	0.064^a^
ALT (U/L), mean (SD)	24.13	18.61	17.61	8.61	0.004^a^
AKP (U/L), mean (SD)	67.4	17.82	69.17	18.25	0.432^a^
GGT (U/L), mean (SD)	25.22	18.19	18.21	7.05	0.001^a^
TC (mmol/L), mean (SD)	4.96	0.7	4.43	0.48	< 0.001^a^
TG (mmol/L), mean (SD)	1.31	1.04	0.98	0.31	0.008^a^
LDL (mmol/L), mean (SD)	2.81	0.51	2.6	0.61	0.001^a^
RBC (× 10^12^/L), mean (SD)	4.77	0.48	4.55	0.46	< 0.001^a^
HGB (g/L), mean (SD)	142.18	16.04	140.98	19.08	0.561^a^
MCHC (g/L), mean (SD)	337.21	6.7	349.71	7.21	< 0.001^a^
HCT, mean (SD)	0.42	0.04	0.4	0.05	0.001^a^

^a^Used two independent-sample t tests, and ^b^ used the chi-square test. *WHR* waist-to-hip ratio, *BMI* body mass index, *AST* aspartate transaminase, *ALT* alanine transaminase, *AKP* alkaline phosphatase, *GGT* gamma-glutamyl transferase, *TC* total cholesterol, *TG* triglycerides, *LDL* low-density lipoprotein, *RBC* red blood cells, *HGB* haemoglobin, *MCHC* mean corpuscular haemoglobin concentration, *HCT* red blood cell specific volume.

### Typical symptom indicators and SV characteristics of subhealth

A comparison of the colour and morphological characteristics of SV between subhealthy and healthy individuals is shown in Table 3. There were statistically significant differences in SV-a, SV-b, tortuosity, and trunk length between the two healthy states (*P* < 0.05). In contrast, the redness of SV in the subhealthy group was paler. In terms of tortuosity, 42.9% of subhealthy individuals showed T2, implying the presence of some degree of sublingual choroidal varicosity. Sixty-eight percent of subhealthy individuals had L1 on the trunk length, which means that the length is shorter than 3/5 of the line between the sublingual caruncle and tip of the tongue. In terms of the width of SV, there was an unprecedented agreement between subhealthy and healthy individuals with a greater concentration on W3, and the maximum width of the vein greater than 4 mm.

**Table 3:** SV characteristics of the physical examinees consisting of 627 subhealthy and 70 healthy individuals; Shanghai, China, 2020–2021.

Characteristics of SV	Subhealthy (n = 627)	Healthy (n = 70)	K/F	*P* value
	N/Mean	N/Mean		
SV-L, mean (SD)	22.20 (4.30)	23.21 (4.15)	1.535	0.061^a^
SV-a, mean (SD)	14.01 (3.11)	17.75 (3.39)	0.279	< 0.001^a^
SV-b, mean (SD)	4.07 (2.13)	6.88 (1.65)	5.716	< 0.001^a^
Tortuosity of SV, N (%)			25.114	< 0.001^b^
T1	196 (31.3)	9 (12.9)		
T2	269 (42.9)	22 (31.4)		
T3	162 (25.8)	39 (55.7)		
Trunk-length of SV, N (%)			37.304	< 0.001^b^
L1	427 (68.1)	25 (35.7)		
L2	109 (17.4)	13 (18.6)		
L3	91 (14.5)	32 (45.7)		
Width of SV, N (%)		25 (35.7)	0.611	0.685^b^
W1	80 (12.8)	10 (14.3)		
W2	71 (11.3)	10 (14.3)		
W3	476 (75.9)	50 (71.4)		

^a^Used two independent-sample t tests, and ^b^Used the Mann‒Whitney U test. *SV* sublingual veins, SV-L the value of the sublingual veins on the L-axis in Lab colour space, SV-a the value of the sublingual veins on the a-axis in Lab colour space, SV-b the value of the sublingual veins on the b-axis in Lab colour space, T1 grade 1 of sublingual veins tortuosity, T2 grade 2 of sublingual veins tortuosity, T3 grade 3 of sublingual veins tortuosity, L1 grade 1 of sublingual veins trunk-length, L2 grade 2 of sublingual veins trunk-length, L3 grade 3 of sublingual veins trunk-length, W1 grade 1 of sublingual veins width, W2 grade 2 of sublingual veins width, W3 grade 3 of sublingual veins width.

**Figure 5:**
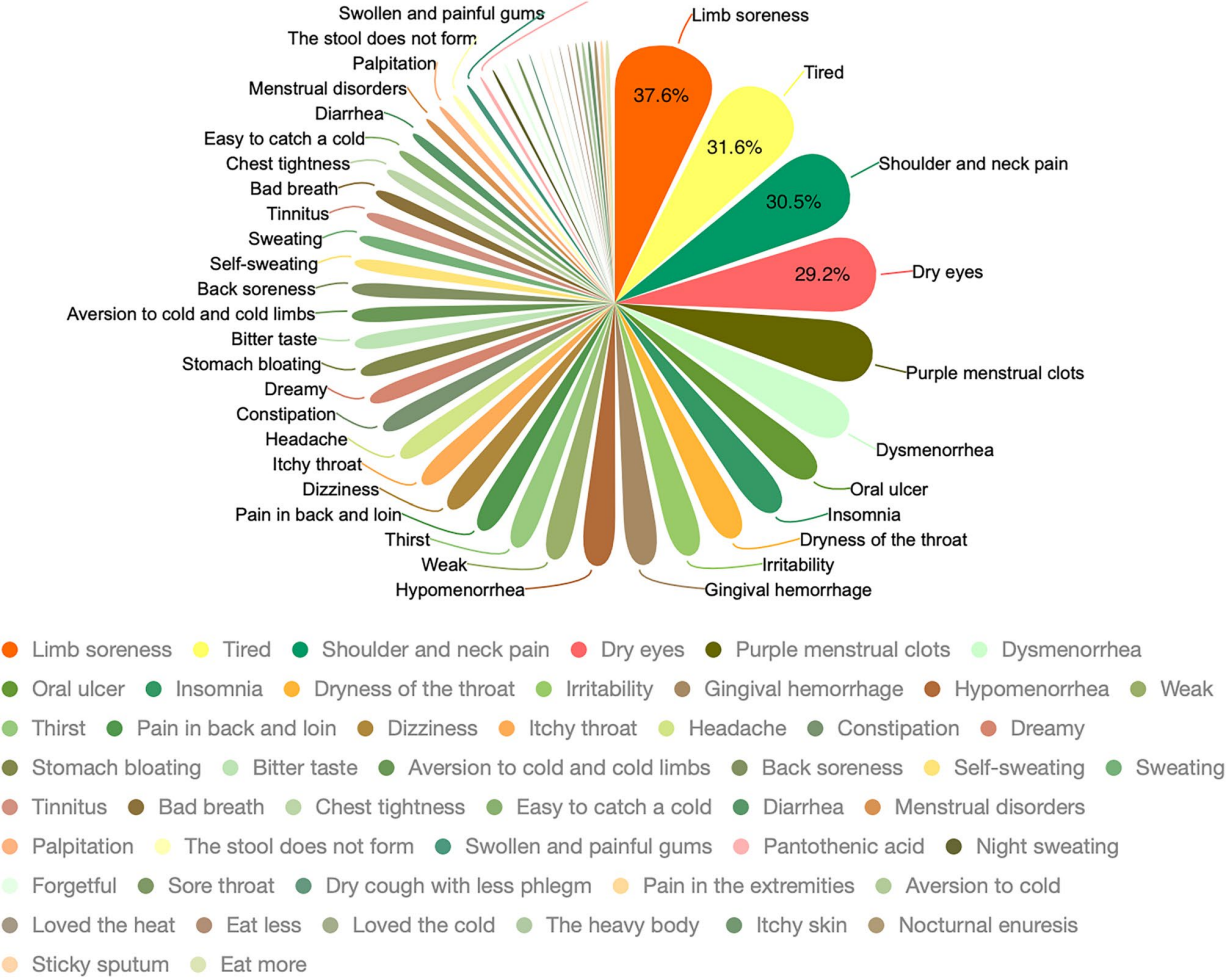
Distribution map of high frequency symptoms in subhealthy individuals (n = 627); Shanghai, China, 2020–2021.

Clinical diagnosis records of TCM are the results of clinical doctors' inquiries, which have important guiding significance for subhealth evaluation. Therefore, this study evaluated all symptoms of TCM clinical diagnosis records and counted the typical manifestations of 627 subhealth examiners according to their degree and frequency. The top 50 high-frequency symptoms are shown in Fig. 5.

The most common manifestations of subhealth were limb soreness (37.6%), fatigue (31.6%), shoulder and neck pain (30.5%) and dry eyes (29.2%). This may be related to the fact that the subhealthy people were younger and had a higher level of education; therefore, they may have been engaged in mental work in an indoor setting, and thus their work habits led to the abovementioned adverse symptoms. In fact, people with a higher educational level are much more likely to be subhealthy than those with a lower educational level, as has been confirmed in another cross-sectional report^32^.

**Figure 6:**
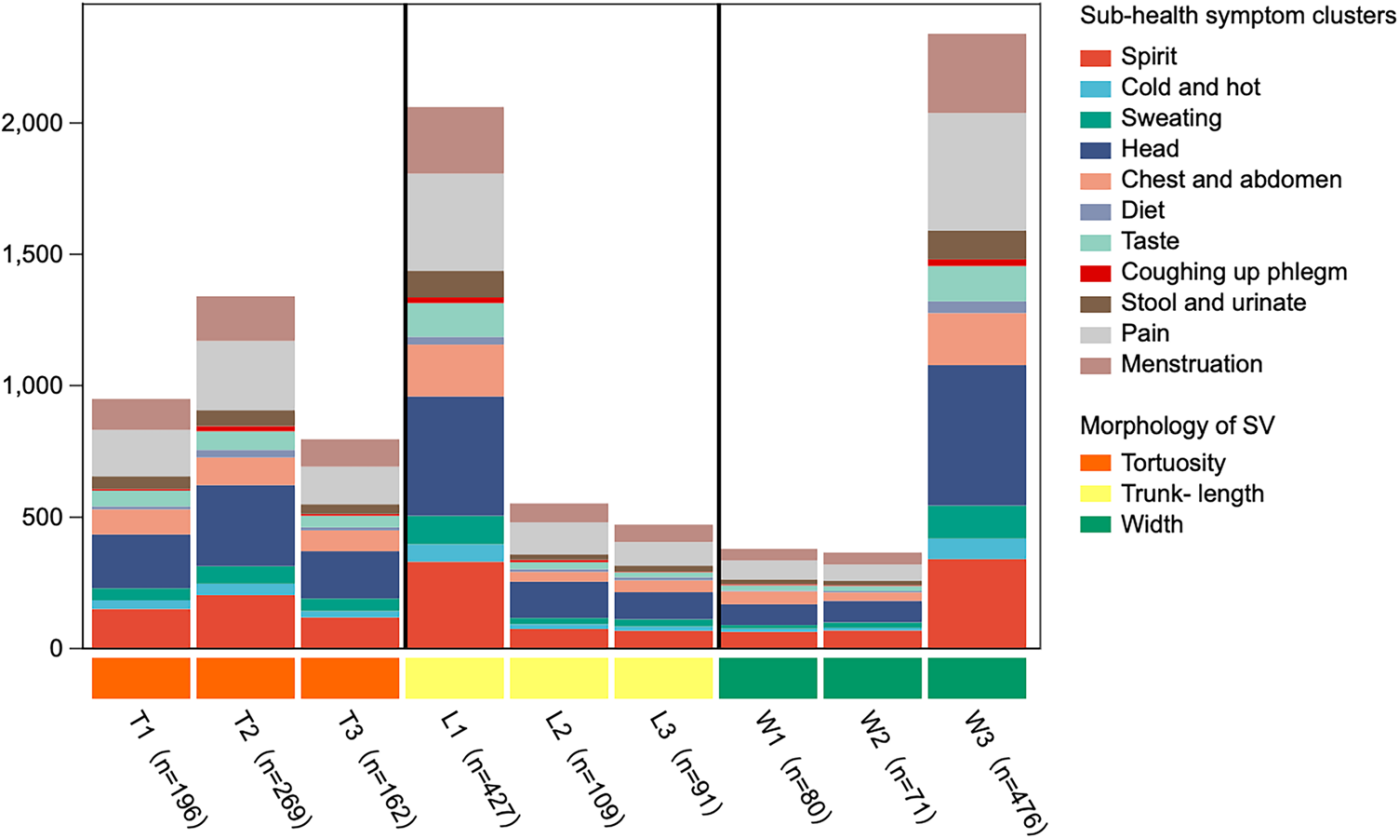
Vertical grouping histogram of subhealthy symptom clusters and morphology of SV; Shanghai, China, 2020–2021. From left to right, the X-axis is the tortuosity (T1, T2, T3) of the SV, trunk length (L1, L2, L3) of the SV and width (W1, W2, W3) of the SV. The Y-axis is the cumulative frequency of symptoms in 11 symptom clusters.

According to the "Ten questions" of TCM^33^ (which are more than 600 years old and originated from the famous ancient doctor Zhang Jingyue's questioning of the patient's symptoms, categorizing a patient’s condition into 10 highly generalized questions, such as fear of cold or heat, sweating or not), these high-frequency symptoms were classified into 11 symptom groups: mental psyche, cold and heat, sweating, head, chest and abdomen, diet, taste, coughing-up phlegm, passing stool and urinating, pain, and menstruation. Combining the foundation of the previous study, we further observed the relationship between subhealth symptom clusters and SV morphology and found that pain, head performance, and psyche were most frequent in subhealthy individuals when the width of the SV was found to be thickened to W3. At the same time, these symptom clusters are also common in subhealthy people with an L1 trunk length of SV (Fig. 6).

### Relationship chains of subhealth physical examination indicators

PLS-SEM demonstrates the structure of a relational chain in which latent variables can be separately represented by a set of observed variables. PLS-SEM comprises a structural model (inner model) that describes the relationship between latent variables and a measurement model (outer model) that describes the relationships among the latent variables and their measurement indicators. In this study, the measurement model consisted of the formative measurement model (for obesity) and the reflective measurement model (for routine blood tests, lipid metabolism, liver function and characteristics of SV). We evaluated the inner model by estimating the path coefficients between latent variables, discriminant validity, significance values, etc., and evaluated the outer model by confirming the reliability and validity of the measurement indicators, ultimately, to verify the reasonableness of the assumed relationship in the model^22^.

Rectangles indicate observed variables, and ovals indicate latent variables. The arrows connecting the ovals and the numbers indicate the paths and path coefficients between the latent variables, whereas the thick black triangular arrows connect the chain of relationships (Supported); otherwise, there is no relationship (Rejected).

**Figure 7:**
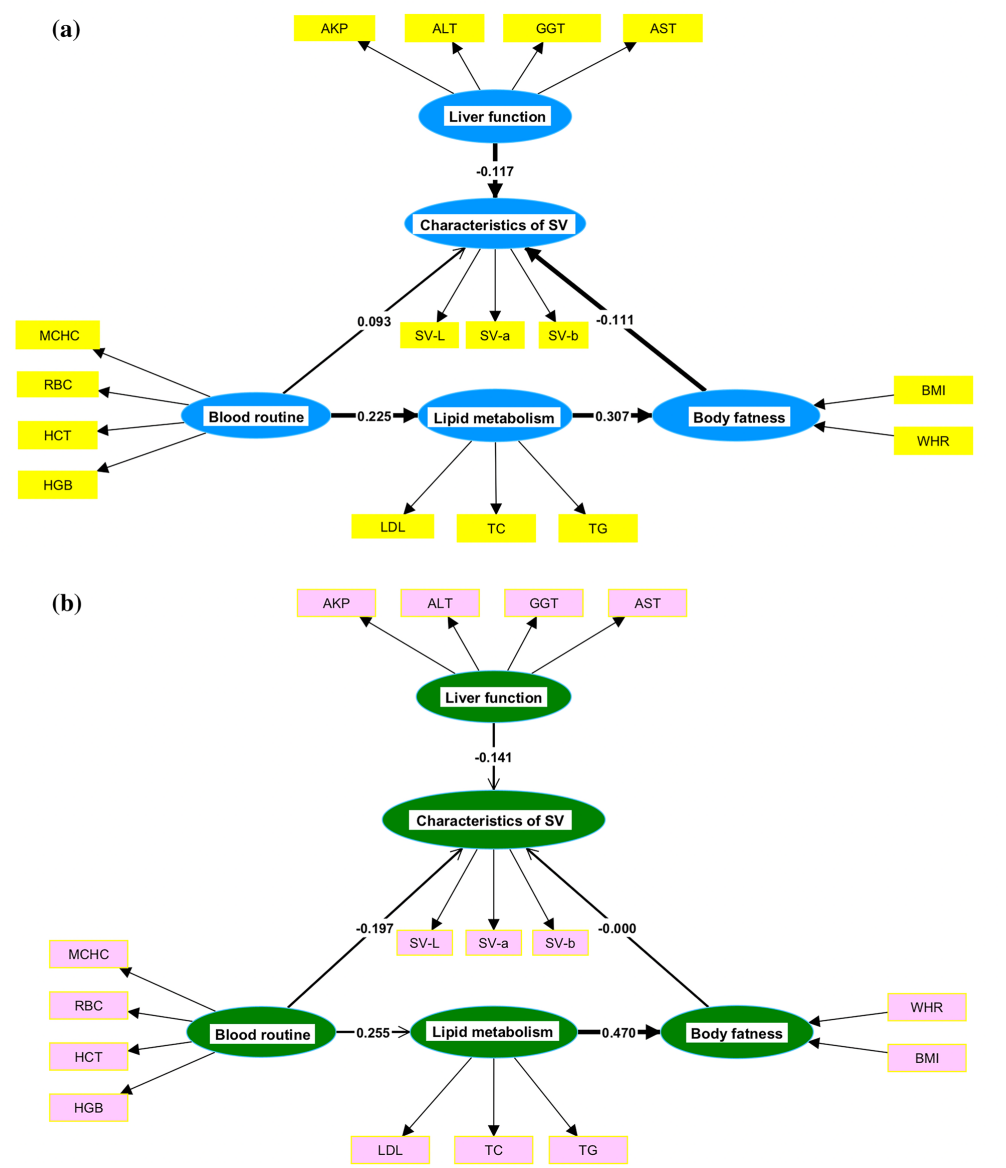
PLS-SEM for estimating the relationship of physical examination indicators; Shanghai, China, 2020–2021. Rectangles indicate observed variables, and ovals indicate latent variables. The arrows connecting the ovals and the numbers indicate the paths and path coefficients between the latent variables, where the thick black triangular arrows connect the chain of relationships (Supported); otherwise, there is no relationship (Rejected). (**a**) PLS-SEM of subhealth with path coefficients, (**b**) PLS-SEM of health with path coefficients.

Through the path relationship between latent variables, we could see that the path between routine blood tests and lipid metabolism, the path between lipid metabolism and obesity, the path between obesity and characteristics of SV and the path between liver function and characteristics of SV were established in Fig. 7a and Table 4. In terms of physical examination indices of healthy people, the path between lipid metabolism and obesity was also established in Fig. 7b (coefficient = 0.470, t = 4.175, *p* < 0.001). Lipid metabolism has a direct effect on body shape in both healthy and subhealthy people.

**Table 4:** PLS-SEM subhealth model path coefficients; Shanghai, China, 2020–2021.

Path	Coefficients	Bootstrapped sample mean (SD)	t	*P* value	Decision
Routine blood test → Characteristics of SV	0.093	0.093 (0.082)	1.133	0.257	Rejected
Routine blood test → Lipid metabolism	0.225	0.231 (0.029)	7.878	< 0.001	Supported
Obesity → Characteristics of SV	− 0.111	− 0.110 (0.049)	2.237	0.025	Supported
Lipid metabolism → Obesity	0.307	0.319 (0.037)	8.410	< 0.001	Supported
Liver function → Characteristics of SV	− 0.117	− 0.126 (0.053)	2.215	0.027	Supported

*SV* sublingual veins.

Table 5 analyses the performance of the PLS-SEM outer model to verify the reasonableness of the subhealth physical examination indicator relationship chain. Cronbach’s alpha ≥ 0.70 and outer weights > 0.20 indicate good reliability and validity of the outer model. AVE ≥ 0.50 and CR ≥ 0.70 indicated good convergent validity and combined reliability of the reflective measurement model, while outer weights > 0.20 and VIF < 5 indicated acceptable validity and indicator appropriateness of the formative measurement model.

**Table 5:** Assessment of PLS-SEM subhealth model; Shanghai, China, 2020–2021.

Construct	Type	Items	Loadings	Cronbach’s alpha/outer weights	AVE/VIF	CR
Routine blood test	Reflective	HGB	1.00	0.86	0.74	0.91
		MCHC	0.57			
		RBC	0.84			
		HCT	0.97			
Lipid metabolism	Reflective	TC	0.88	0.69	0.61	0.82
		TG	0.74			
		LDL	0.72			
Liver function	Reflective	AKP	0.70	0.75	0.56	0.83
		ALT	0.80			
		AST	0.72			
		GGT	0.78			
Characteristics of SV	Reflective	SV-L	0.64	0.71	0.64	0.84
		SV-a	0.95			
		SV-b	0.78			
Obesity	Formative	BMI	0.91	0.71	1.23	–
		WHR	0.77	0.46	1.23	

*AVE* average variance extracted, *VIF* variance inflation factors, *CR* composite reliability, *SV* sublingual veins, *HGB* haemoglobin, *MCHC* mean corpuscular haemoglobin concentration, *RBC* red blood cells, *HCT* red blood cell specific volume, *TC* total cholesterol, *TG* triglycerides, *LDL* low-density lipoprotein, *AKP* alkaline phosphatase, *ALT* alanine transaminase, *AST* aspartate transaminase, *GGT* gamma-glutamyl transferase, *BMI* body mass index, *WHR* waist-to-hip ratio.

Table 6 assesses the PLS-SEM inner model and reveals the correlations between latent variables and the heterotrait–monotrait ratio. In the inner model, HTMT < 0.85 and correlation value < 0.70 both indicate that the constructs have some discriminant validity.

**Table 6:** Discriminant validity (HTMT) and correlations among latent variables; Shanghai, China, 2020–2021.

	Routine blood test	Lipid metabolism	Liver function	Characteristics of SV	Obesity
Routine blood test	**1.000**	0.225	0.468	− 0.002	0.365
Lipid metabolism	0.275	**1.000**	0.253	− 0.058	0.307
Liver function	0.564	0.323	**1.000**	− 0.126	0.471
Characteristics of SV	0.144	0.091	0.159	**1.000**	− 0.132

When the correlation value between latent variables is equal to 1, it is bolded on the diagonal, above which are the correlations among the construct values and below which are the HTMT values. SV sublingual veins.

## Discussion

In this study, the PLS-SEM was applied to subhealth for the first time. The relationship between subhealth examination indices was studied in the form of the directed acyclic relationship chain^34^. This study validates the establishment of the relationship chain: the path from routine blood tests to lipid metabolism, the path from lipid metabolism to obesity, the path from obesity to characteristics of SV, and the path from liver function to characteristics of SV. Similar methods have been used to study the relationship between variables. Ramirez et al.^35^ studied the association between atopic dermatitis and sleep duration and quality among children using a directed acyclic graph. Woojin Kim et al.^36^ used a PLS-SEM approach to reveal that impaired glucose metabolism caused by obesity affects memory decline as well as regional grey matter atrophy in elderly individuals with no neurological disease. This is precisely because the clinical manifestations of subhealth are complex and difficult to quantify. It is necessary to skilfully use the structural relationship model to carry out objective research from physical examination indicators, which greatly makes up for the subjective defects of previous subhealth research. It also contributes to the promotion of the concept of subhealth all over the world.

The previous surveys of subhealthy people are based on large samples, but they rarely go beyond a simple correlation analysis of indicators and explore the potential relationship between the main physical examination items from a macro point of view. The relationship chain of subhealth physical examination indicators derived from this study is not only consistent with clinical practice but also provides a basis for deeper excavation of subhealth mechanisms and causal inference.

The relationship chain between lipid metabolism and obesity is established in both subhealthy and healthy people. Abnormal lipid metabolism can cause changes in physical characteristics, which is widely recognized in clinical practice and epidemiological research. There was also a significant correlation between the two in another large sample population survey^11^.

To explain the path from obesity to characteristics of the SV, previous studies have found that SV are the accompanying veins of the hypoglossal nerve and lingual nerve in the lamina propria and sublayer of the ventral mucous membrane of the tongue. The mucosa on the ventral surface of the tongue is smooth and thin, and the mucosal epithelium is a stratified flat epithelium with neither tongue papillae nor keratosis^37–40^. Compared with the tongue surface covered with keratinized epithelium, mucosal epithelium, secondary papillae and exudate, SV can more sensitively reflect the state of the body. There are obvious physiological differences among individuals of different ages and sexes in SV characteristics^18^. Therefore, as one of the manifestations of physiological differences among different individuals, obesity is also reflected in the characteristics of SV.

To explain the path from liver function to characteristics of SV, the concept of "liver tongue" has long appeared in TCM, and it is considered to be a specific manifestation of liver cirrhosis^41^. A study recommended that the measurement of tongue thickness should be considered for diagnosing sarcopenia in cirrhosis of the liver^42^. Another study found that patients with liver cirrhosis are more likely to develop sublingual varices^43^, specifically swelling and extreme fullness of the SV. This study further demonstrates the relationship between them, and it may be a convenient and effective method to explore the changes in SV in the process of judging the development and prognosis of liver disease.

In addition to the above relationship chain, we also found the relationship chain between routine blood tests and lipid metabolism. We can further explore their role in subhealth mechanisms in the future.

In the past, few studies on subhealth were combined with TCM diagnostic data. In this study, the clinical data of TCM and Western medicine were fused, and inquiry into TCM and tongue diagnosis characteristics was creatively added. The tongue has traditionally been seen as a mirror for the internal organs, reflecting the body’s physiological and clinical-pathological condition^44^. Incorporating TCM tongue features, including the SV, into the physical examination can help to comprehensively assess the current state of subhealth and further predict the transformation of subhealth. It was found that the typical manifestations of subhealthy people were limb soreness, tiredness, shoulder and neck pain and dry eyes. The subhealthy symptom group was closely related to SV status. The typical symptoms of subhealth in this study are basically consistent with a previous survey of the fatigued subhealthy population^22^. Understanding the typical manifestations of subhealth can improve people's understanding of physical function^45^ and promote further targeted examination and lifestyle intervention for risk groups^5^. It is the embodiment of the global public health concept of progressing the treatment of diseases and giving priority to prevention.

### Limitations of the study

There were some limitations in this study. First, due to the accessibility of the data, this study was single-centre, and the physical examination population entering this cross-section was generally younger, which may lead to some restrictions on the representativeness and feasibility of the research results. Second, this cross-sectional study analysed the TCM diagnostic data and Western physical examination indicators of subhealthy people. However, there was a lack of eating habits and exercise habits closely related to subhealth. If the above information can be included, the assessment of subhealth will be more complete and accurate.

## Conclusions

In this study, PLS-SEM was creatively applied to the relationship of subhealth physical examination indices, and it was found that the path from routine blood tests to lipid metabolism, the path from lipid metabolism to obesity, the path from obesity to characteristics of SV and the path from liver function to characteristics of SV were reasonable. At the same time, combined with the diagnostic methods of TCM, we found that the typical manifestations of subhealth were limb pain, tiredness, shoulder and neck pain and dry eyes, which could provide an idea for simplifying physical examination items and reducing medical expenses for subhealthy people in the future. The targeted regulation and treatment of adverse symptoms will be expected to advance the treatment of diseases and improve the national health level.

### Supplementary Information

Supplementary Table S1.

## Data Availability

The datasets generated and analysed during the current study are not publicly available due to the confidentiality of the data, which is an important component of the National Key Technology Research and Development Program of the 13th Five-Year Plan (No. 2017YFC1703301) in China, but they are available from the corresponding author on reasonable request.

## Abbreviations

PLS-SEM: Partial least squares-structural equation modelling
SV: Sublingual veins
HGB: Haemoglobin
RBC: Red blood cells
TC: Total cholesterol
TG: Triglycerides
ALT: Alanine transaminase
AST: Aspartate transaminase
BMI: Body mass index
WHR: Waist-to-hip ratio
H20: Health Status Assessment Questionnaire
AKP: Alkaline phosphatase
GGT: Gamma-glutamyl transferase
LDL: Low-density lipoprotein
MCHC: Mean corpuscular haemoglobin concentration
HCT: Red blood cell specific volume
TCM: Traditional Chinese medicine
TFDA-1: Tongue and face diagnosis instrument-1
TDAS: Tongue diagnosis analysis system
